# Marine-derived *Pseudomonas gessardii* E2 strain: a novel source of green fluorescent protein with insights into its optimization and purification

**DOI:** 10.1186/s12934-026-02960-9

**Published:** 2026-03-11

**Authors:** Fafy A. Mohammed, Hend A. Anter, Sherif Moussa Husseiny

**Affiliations:** Botany Department, Faculty of Women For Arts, Science and Education, Ain Shams University, Cairo, Egypt

**Keywords:** Fluorescent-bacteria, Green fluorescence protein, Vitek 2 system, *Pseudomonas gessardii*, Aqueous two-phase system, Fast protein liquid chromatography

## Abstract

**Background:**

Green fluorescent protein (GFP) is a valuable macromolecule widely utilized in biomedical diagnostics and diverse biotechnological applications due to its low toxicity and intrinsic fluorescence, which does not require exogenous substrates or cofactors. Nevertheless, the conventional *Aequorea victoria*–derived GFP exhibits limitations, including relatively low brightness and restricted applicability under low-oxygen conditions. Consequently, there is increasing interest in identifying alternative and less explored microbial sources of GFP, particularly from marine environment.

**Results:**

In the current study, eleven fluorescent bacteria were isolated from different anatomical parts of squid collected from Miami Beach (Alexandria, Egypt) and from seawater sample from Atubia island (Safaga, Egypt). Among the three isolation media evaluated, Sea Water Complete agar medium (SWC) supported the highest recovery of fluorescent bacteria. Fluorescence-based primary and secondary screening identified isolate E2 as the most efficient GFP producer. It was identified phenotypically using the VITEK^®^ 2 automated system and genotypically using 16 S rRNA studies as *Pseudomonas gessardii* with 99.34% similarity. Sequences of the 16 S rRNA gene were deposited in the GenBank as OQ285875. Optimization of environmental condition using a one-factor-at-a-time approach revealed the maximum GFP production was achieved after 30 h of incubation at 25 °C and pH 7.5 under static conditions (0 rpm). These optimized parameter enhanced the fluorescence intensity of GFP by 1.51-fold compared to the basal medium. Subsequent purification using an aqueous two-phase system and fast protein liquid chromatography successfully recovered GFP with a high purity of 96.3%.

**Conclusion:**

Overall, the current study identifies *Pseudomonas gessardii* E2 strain as a novel marine-derived source of high-quality pure GFP and establishes an effective strategy for its production and purification. The findings provide a promising foundation for the potential application of this GFP in bioimaging, biosensing, and fluorescence-based reporter systems, including applications in oxygen-limited environments.

**Supplementary Information:**

The online version contains supplementary material available at 10.1186/s12934-026-02960-9.

## Background

 Biofluorescence is the phenomenon whereby a portion of a living beings absorbs electromagnetic radiation at one wavelength then emits light at longer wavelength, glowing with green, blue, and red bright fluorescent colors. Biofluorescence is invisible by the unaided eye and can only be detected with a UV light source operating in the 320–390 nm range, with 365 nm being the most commonly used, and corresponding emissions observed within 450–550 nm range [1]. The mechanisms by which biofluorescent light being produced are diverse and involve different proteins such as GFP, pigments, metabolites, or even mineralization. The functions of biofluorescence in nature include communication, mate selection, camouflage, enhanced visual perception, or, in some cases, it may serve no apparent biological purpose [2]. The exploration of biofluorescence first evolved in 1962 with the discovery and subsequent applications of GFP isolated from the *Aequorea victoria* jellyfish [3]. The native GFP consists of 238 amino acids with approximately 27 kilodaltons (kDa) molecular weight. It exhibits two primary peaks of absorption; the first major one at around 395 nm representing the neutral state of the chromophore (protein portion responsible for energy absorption and light emission), and a minor peak at 480 nm associated with its deprotonated anionic form, while its emission maximum occurs at approximately 508 nm. The GFP chromophore is consisted by specific amino acid residues (Ser65-Tyr66-Gly67) and is protected from quenching by water through its location within an α-helix surrounded by 11 β-barrel sheets [4]. Unlike many fluorescent reporters, GFP autofluoresces without the need of any exogenous substrate or cofactors. Moreover, GFP exhibits negligible toxicity, a high degree of resistance to denaturation (melting temperature of 78 °C) and proteases, stability throughout a broad pH range (5–12), and retention of its activity following fixation with other liquids, rendering it highly advantageous in live-cell imaging applications [5, 6]. Consequently, GFP has been used extensively as protein marker for monitoring gene expression, protein mapping for tracking cellular processes in living organisms [7], and also is a promising component for biosensor and biomarker applications in biotechnology [8]. Despite these extensive uses, *A. Victoria*-derived GFP exhibits several inherent limitations that restricted its performance in certain applications. These include reduced photostability that limits its usefulness in fluorescence microscopy, resulting in notable photobleaching during imaging sessions [9], relatively low brightness, and a noticeable maturation delay between protein synthesis and the development of noticeable fluorescence [10]. Moreover, GFP chromophore is strictly dependent on the presence of molecular oxygen, which limits its application in low-oxygen environments, such as tumor microenvironment, ischemic tissues, or research involving obligate anaerobic organisms [11]. These limitations have driven ongoing efforts to identify alternative GFP sources with improved functional characteristics. Marine bacteria represent a promising and largely unexplored reservoir of novel GFP variants, as they have evolved under saline and environmentally fluctuating conditions that may confer enhanced stability, expression efficiency, and functional robustness. These microbes live in a variety of oceanic habitats, including sediments and water columns, and they also form symbiotic relationships with fish and squid [12, 13]. Notably, squids have a specialized unique ink sac containing fluorescent microorganisms that work in tandem with them as a protection mechanism [14]. Several genera such as *Shewanella*, *Vibrio*, *Photobacterium*, and certain *Pseudomonas* species are well-known for their bioluminescence or natural fluorescence [15].

*Pseudomonas gessardii* is gram negative bacteria belongs to the Pseudomonas genus, which is renowned for its metabolic adaptability and ecological diversity. *P. gessardii* belongs to the *P. fluorescens* group and is further categorized within the *P. gessardii* subgroup. The genus is taxonomically complicated and contains more than 340 validly recognized species. *P. gessardii* have primarily isolated from different environments, including soil, plants, fish, and aquatic habitats [16, 17].

The production of several microbial proteins is influenced by various physical factors, notably temperature, pH, and incubation period, which affect not only protein yield but also stability, aggregation, and functionality of proteins during both submerged liquid and solid state fermentation [18, 19]. In the case of GFP, these physical factors also influence its photophysical behavior [20]. For large scale application, high concentrations of purified GFP are often required to overcome background autofluorescence which can interfere with the detection and quantification of GFP signals in biological systems. Therefore, optimizing GFP production and efficient purification protocol is highly required for large-scale application [21]. Therefore, the present study focuses on isolating a novel and practical marine-derived source of GFP, optimizing culture conditions for enhanced protein yield, and establishing of effective purification steps to support potential applications in bioimaging, biosensing, and fluorescence-based reporter systems.

## Methods

### Sampling and bacterial isolation

Three freshly caught marine squids were obtained early in the morning from local fisherman at Miami beach, Alexandria, Egypt, in December 2022. Samples were kept in a half-submerged icebox with sea water at 18 °C and then immediately brought to the research laboratory. The squid specimens were gently rinsed with distilled water to get rid of any adhering sand or debris and were kept cold to impair the growth of any unwanted bacteria and prevent decomposition. Marine water sample was collected from Atubia Island (26° 49’ 49.6" N, 33° 58’ 42.87" E), Safaga, Red Sea, Egypt, at 14 m depth and 2.4 km from the beach. Water parameters, including temperature, pH and electrical conductivity, were measured via the portable hydrolab. The water sample was collected in sterile 250 ml bottles and kept at 4 °C until processing in the laboratory [22]. Three different isolation media were employed: Seawater Complete agar (SWC) [23], Luminescent medium (LM) [24], and Boss medium [25]. Several anatomical parts of the squid, including eyes, gills, skin, gut and tentacles, were swabbed and streaked onto the surface of the isolation media and incubated at 20 °C for 72 h [26, 27]. The intestinal region was aseptically dissected to extract the ink sac. One milliliter of the ink was aseptically transferred to Falcon tube containing 9 ml of luminescent broth to prepare a 10⁻¹ dilution, followed by serial dilutions up to 10⁻⁵. Aliquots (0.1 ml) from each dilution and the seawater sample were spread onto the different three media and incubated under the same conditions [28].At the end of incubation period, the plates were examined for fluorescent bacterial colonies using MOPEL ENF-260 C UV lamp at 365 nm (MOPEL instruments, New York, U.S.A) in the dark. Fluorescent colonies were carefully picked and re-streaked onto fresh isolation media to obtain pure cultures.

### Selection of the best growth medium for the growth of fluorescent bacteria

To determine the most suitable medium supporting fluorescent bacterial growth, a standardized bacterial suspension (10^8^ CFU/ml) of each isolate was diluted to obtain to obtain concentration ranged from 10^− 1^ to 10^− 5^. 10^− 4^ dilution was then plated on to the surface of the three isolation agar media. Following incubation at 20 °C for 24 h., the medium yielding the highest total viable count for all isolates was selected for the culturing of fluorescent bacteria [29].

### Screening for fluorescence production

An inoculum (5 ml; 10⁸ CFU/ml) of each fluorescent bacterial isolate was cultured in 100 ml SWC broth at 20 °C for 24 h. Initial screening of all bacterial isolates was performed through visual assessment under UV illumination at 395 nm in a dark room [12]. Isolates exhibiting the highest visible fluorescence were subjected to secondary screening using a luminescence spectrometer (Perkin Elmer LS-50B, USA) at excitation wavelength of 395 nm to measure fluorescence spectra between 420 and 650 nm. Emission intensity was expressed in arbitrary unit (au) [30].

### Selection of the most potent GFP producing bacterial isolate

The selection was based on the quantification and purity assessment of green fluorescent protein (GFP) from bacterial isolates selected from secondary screening.

#### I. Bacterial protein extraction

Total protein extraction from the selected fluorescent isolates was carried out following the procedures of Meglič et al. [31] with minor adjustments. Bacterial pellets obtained after centrifugation for 15 min at 4000 rpm at 4 °C were suspended in Tris–HCl buffer at pH 7.0 (1:4, w/v). Cells were sonicated on using 20 cycles of 30-s pulses at 20% amplitude, with 30-s intervals to prevent thermal denaturation. Following sonication, the cell lysate was centrifuged once more for 15 min at 4000 rpm at 4 °C, and the resulting filtrate was used as the source of crude soluble protein.

#### II. Estimation of total soluble protein content

Total soluble protein concentration was assessed following the Bradford assay (Sigma-Aldrich, Cat. No. B6916) in accordance the manufacturer’s guidelines. A standard curve was generated employing bovine serum albumin (BSA) within a concentration range of 0.1 to 1.4 mg/ml. Absorbance readings were obtained at 595 nm using a UV–Vis spectrophotometer, and protein concentrations were derived from the corresponding calibration curve [32].

#### III. Sodium dodecyl sulfate polyacrylamide gel electrophoresis (SDS–PAGE) analysis

Soluble protein filtrates were subjected to SDS–PAGE using a resolving gel (12%) according to Ebraheem et al. [18] using BlueAQUa™ Prestained Protein Ladder (Cat No. PM019-500,GeneDirex, Taiwan, 10–180 kDa) as a molecular weight marker.

#### IV. GFP quantification and purity assessment

The bands corresponding GFP approximately at 27 kDa was carefully excised from the gel and eluted using a Protein Extraction Kit from Gel Slices (101Bio, Cat. No. BL-P519-Gp) [33]. Bradford assay was used to determine the concentration of the eluted GFP, while its purity was expressed as the ratio between the GFP concentration and the total soluble protein content in each sample [34]. The bacterial isolate exhibiting the highest GFP concentration and purity was selected as the most potent GFP producer.

$$\begin{gathered} {\text{GFP purity}}\left( {\% } \right) \hfill \\ \quad =\frac{{{\text{GFP concentration}}}}{{{\text{Total protein concentration}}}} \times 100 \hfill \\ \end{gathered} $$  

### Identification of most potent fluorescent bacteria

#### I. Morphological and biochemical characteristics

The morphological characters of the most potent fluorescent bacteria were evaluated based on colony features, including shape, margin, elevation, size, texture, pigmentation, and overall appearance. The microscopic characters were studied using gram staining using a light microscope. Bacterial motility was assessed using Hanging Drop Method [35] and confirmed using semisolid deep agar [36]. The type of flagella was observed using Transmission electron microscope (JEOL JEM 1200 EXII) following Hoeniger [37].

Biochemical profiling of the selected isolate was carried out using the VITEK^®^ 2 automated system, which evaluates 64 biochemical reactions [38].

#### II. Molecular identification

Genomic DNA was isolated from the most efficient fluorescent bacterial strain using DNeasy^®^ Plant Mini kit (Cat.No.69104, Qiagen, Germany) following the manufacturer’s guidelines. Two universal bacterial primers (5´-AGAGTTTGATCITGGCTCAG-3´,forward and 5´-ACGGITACCTTGTTACGA CTT-3´, reverse ) were used for amplification of 16 S rRNA gene of the selected bacteria following the protocol described by Aneja [35]. The PCR amplicons were separated on a 1% agarose gel and 1 Kb DNA ladder (Thermo Scientific, USA) was used to verify the size of expected fragment. The purified PCR product was sequenced using an automated DNA sequencing system (model 3730XL, Applied Bio Systems, USA). Obtained sequences were analyzed for similarity by performing BLASTN (Basic Local Alignment Search Tool) searches against the NCBI GenBank database to determine sequence similarity and closest phylogenetic relatives [39].

Alignment of the sequence with sequences of similar strains was performed using BioEdit software (version 7.0.4.1) for phylogenetic analysis. The phylogenetic tree was generated in MEGA version 11 utilizing the Maximum Likelihood approach. Bootstrap support values were calculated following Felsenstein’s method with 1,000 replicate [40].

### Optimization of physical factors using one-factor-at-a-time strategy (OFAT)

In OFAT strategy, a single variable was altered at a time while all other factors remained constant. Different physical factors were investigated in triplicates ; incubation period (0, 6, 12, 18, 24, 30 and 36 h), agitation speed (0, 100 and 200 rpm), incubation temperature (15, 20, 25, 30 and 35° C), and initial pH (6, 6.5, 7, 7.5, 8, 8.5, and 9) respectively. Experiments were conducted using SWC broth as the basal medium inoculated with 5 ml of 10⁸ CFU/ml bacterial suspension [20]. Following incubation, bacterial growth was assessed by determining cell density at 600 nm, and fluorescence emission intensity was simultaneously measured.

### Statistical analysis

All data were statistically analyzed using CoStat statistical package (v6.4; CoHort Software, Monterey, CA, USA). Differences among means were analyzed through one-way ANOVA, followed by Duncan’s Multiple Range Test to detect significance at *p* < 0.05.

### Application of aqueous two-phase system

GFP was purified following the aqueous two-phase system (ATPS) protocol [41]. In brief, 200 ml of the selected isolate broth culture incubated under optimum conditions was cooling centrifuged for 20 min at 6000 rpm to obtain soluble proteins. Total protein content was determined using Bradford assay [32]. A mixture of 60 ml soluble protein, 18 ml of 5 M NaCl, and 139.8 ml of ammonium sulfate (64% saturated, pH 7.8) was prepared and incubated on ice for 1 h. Precipitated proteins were removed by centrifugation (6000 rpm for 20 min at 4 °C). The supernatant was combined with anhydrous ethanol in a 1:3 (v/v) ratio and shaken for 30 s then centrifuged for 7 min at 4000 rpm. The resulting upper ethanol layer was visualized under UV light (395 nm) to detect GFP then immediately collected to prevent protein denaturation. To this ethanol extract, n-butanol was added at a 1:4 (v/v) ratio and mixed for 30 s then centrifuged (4000 rpm for 7 min at 4 °C) to result two phases, GFP located in the lower aqueous layer. This layer was dialyzed to eliminate residual solvents and small contaminants against 20 mM Tris-HCl buffer (pH 7.8) supplemented with 150 mM NaCl using a 300 kDa cut-off membrane [18].

### Fast protein liquid chromatography (FPLC)

Further purification of GFP was carried out using an ÄKTA Avant 150 chromatography system (GE Healthcare Life Sciences, USA). UNICORN^®^ software version 6.3 was used for managing the process [34]. The dialyzed GFP fraction was equilibrated with binding buffer of 10 mM Tris–HCl, 10 mM EDTA, and 1.7 M ammonium sulfate at pH 8.0 then loaded onto a 1 ml HiTrap ™ Butyl FF 1 ml HIC (Hydrophobic interaction column). Elution of bound GFP was achieved using a linear gradient of buffer of 10 mM Tris–HCl and 10 mM EDTA at pH 8.0 with a flow rate of 1 ml/min. GFP was monitored in each fraction using UV detector at 365 nm. Fluorescent fractions were collected then dialyzed against 20 mM Tris–HCl buffer at pH 7.8.

### Molecular weight determination and spectral properties of purified GFP

SDS-PAGE (12%) was employed to confirm GFP purity and estimate its molecular weight, utilizing BLUeye™ Prestained Protein Ladder (GeneDirex, Taiwan; 10–245 kDa) as a molecular weight marker [18]. The GFP band was excised and recovered from the gel using a Protein Extraction Kit from Gel Slices (101Bio, Cat. No. BL-P519-Gp) for concentration measurements [33]. The emission spectrum of purified GFP was measured between 420 and 620 nm using a luminescence spectrometer (Perkin Elmer LS-50B, USA) at excitation wavelength of 395 nm.

## Results and discussion

### Recovery and purification of fluorescent producing bacteria

**Fig. 1:**
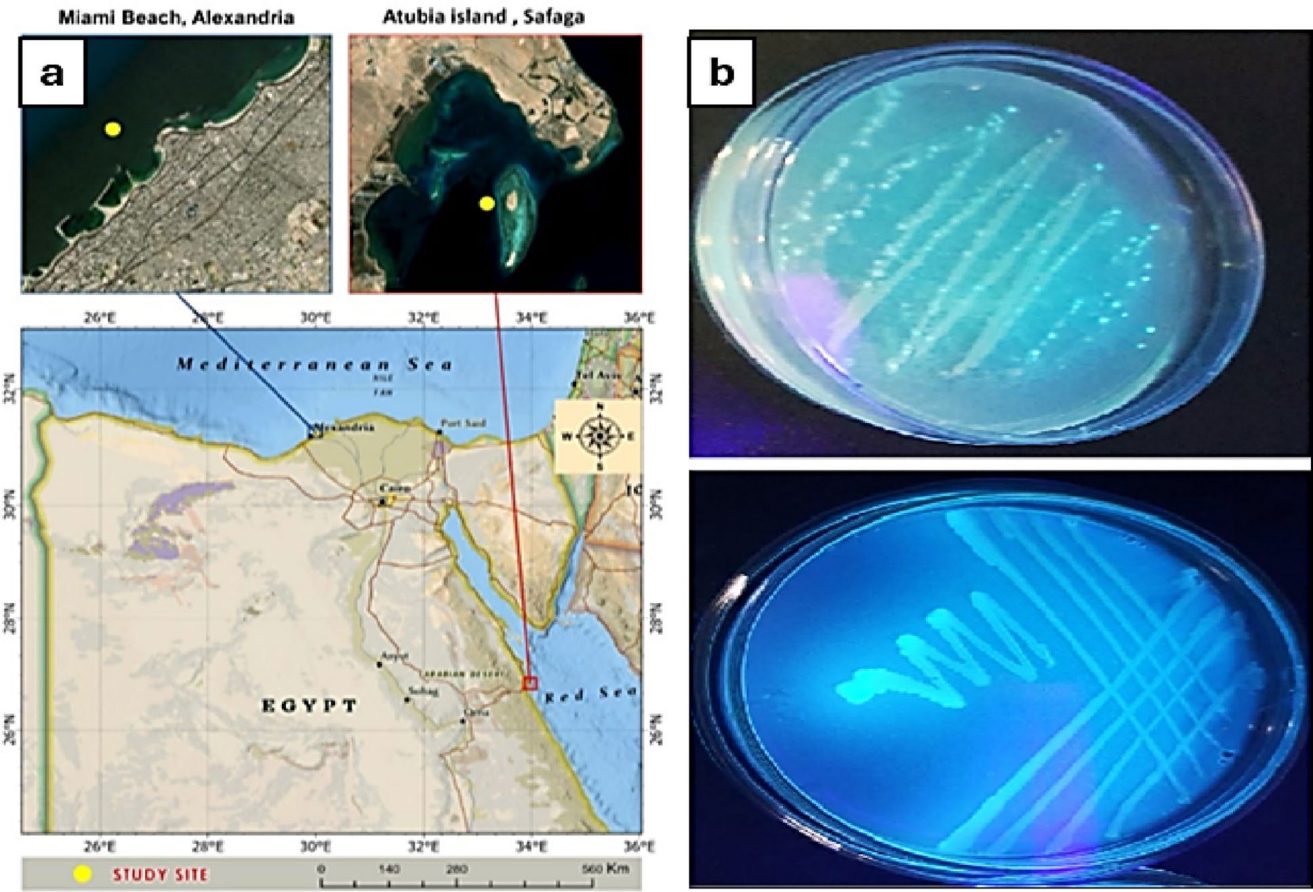
(**a**) Sampling locations at Maimi, Alexandria and Atubia Island, Red sea, Egypt. (Provided by National Authority for remote Sensing and Space Sciences (NARSS), Cairo, Egypt. (**b**) Fluorescent bacteria on isolation media under UV lamp at 390 nm

A total of eleven fluorescent bacteria were successfully recovered from marine squid tissues and seawater sample. Squid-derived isolates were recovered from ink sac (I1, I2), eyes (E1, E2), gills (G1), and gut (Gu1, Gu2), whereas no fluorescent colonies were recovered from the tentacles or skin tissues. Additionally, four fluorescent isolates (S1-S4) were obtained from seawater sample collected from Atubia Island in the Red Sea. The geographical locations of samples collection are illustrated in Fig. 1a, while representative isolates exhibiting fluorescence under UV illumination are displayed in Fig. 1b. Physicochemical analysis of the seawater sample showed a slightly alkaline pH (7.6) and high electrical conductivity (24,448 µS/cm), indicating elevated salinity with sodium and chloride as the dominant ions (Table 1). These characteristic marine conditions are known to support the growth of marine fluorescent bacteria.

**Table 1:** Physicochemical parameters of the seawater sample collected from Atubia Island, Red Sea, Egypt

Parameters	Unit	Concentration
pH		7.6
Total dissolved solids (TDS)	ppm	15.647
Electrical conductivity (EC)	µS/cm	24,448
Sodium (Na^+^)	Meq/L	344.80
Potassium (K^+^)	Meq/L	1.12
Calcium (Ca^2+^)	Meq/L	13
Magnesium (Mg^2+^)	Meq/L	23.08
Chloride (Cl^−^)	Meq/L	350
Bicarbonate (HCO_3_^−^)	Meq/L	12.7
Sulphate (SO_4_^2−^)	Meq/L	19.3

### Selection of growth medium supporting the growth of fluorescent bacteria

**Fig. 2:**
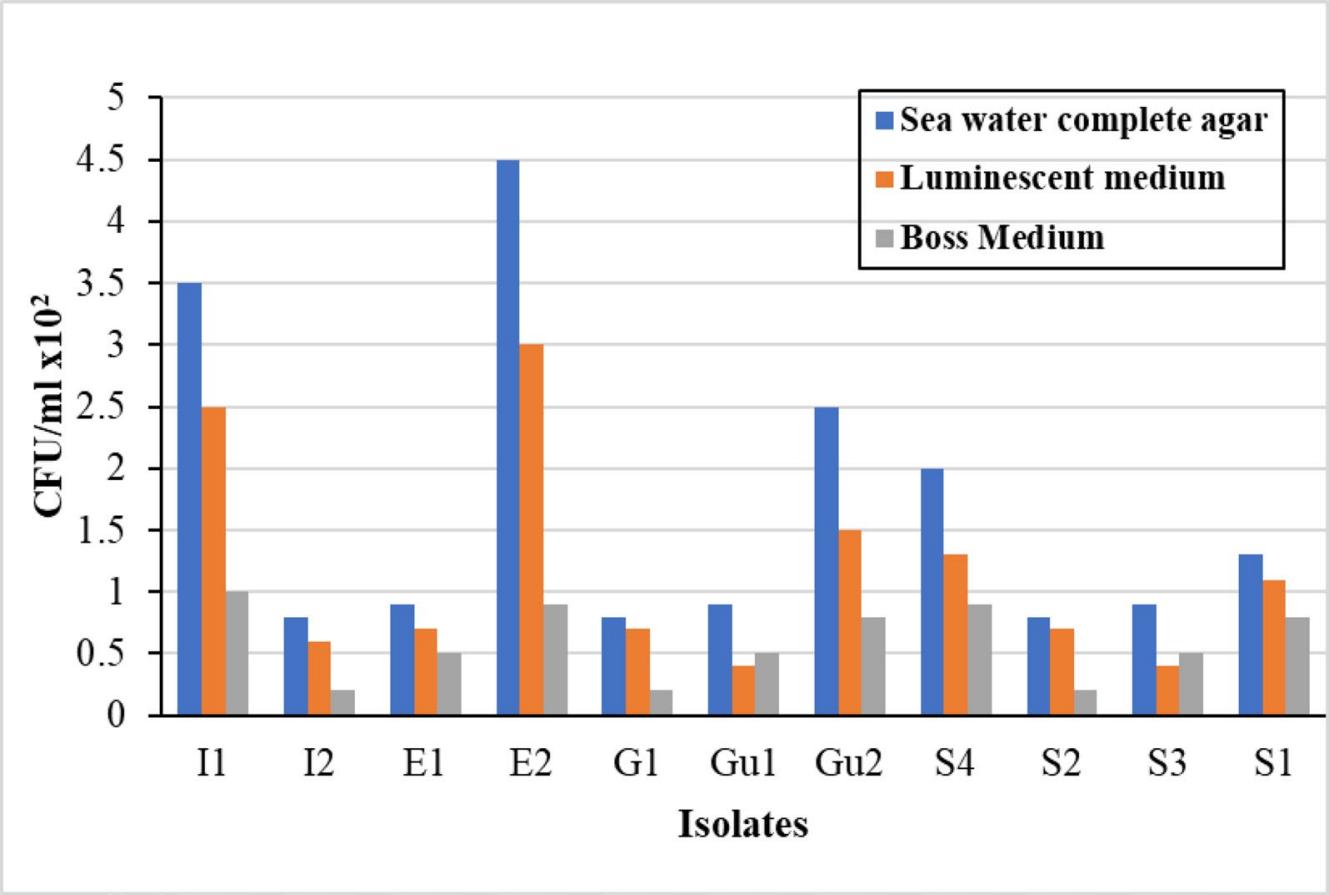
Viable count of fluorescent bacteria isolated from squid parts and sea water sample on different media

Comparative evaluation of the tested isolation media revealed that SWC agar supported the highest viable count of fluorescent bacteria, followed in descending order by Luminescent Medium, then Boss Medium (Fig. 2). SWC agar medium was recommended by Balan et al. [29] for recovery of luminescent bacteria isolated from squid and cuttle fish. In addition, Bayyana et al. [14] and Parmar et al. [42] recorded SWC medium as the most suitable medium supporting luminescence bacterial growth. Conversely, Dubey and Sharon [43] recommended BOSS medium for luminescent bacteria isolation from ink sac of squid.

### Screening of GFP-producing bacterial isolates

**Fig. 3:**
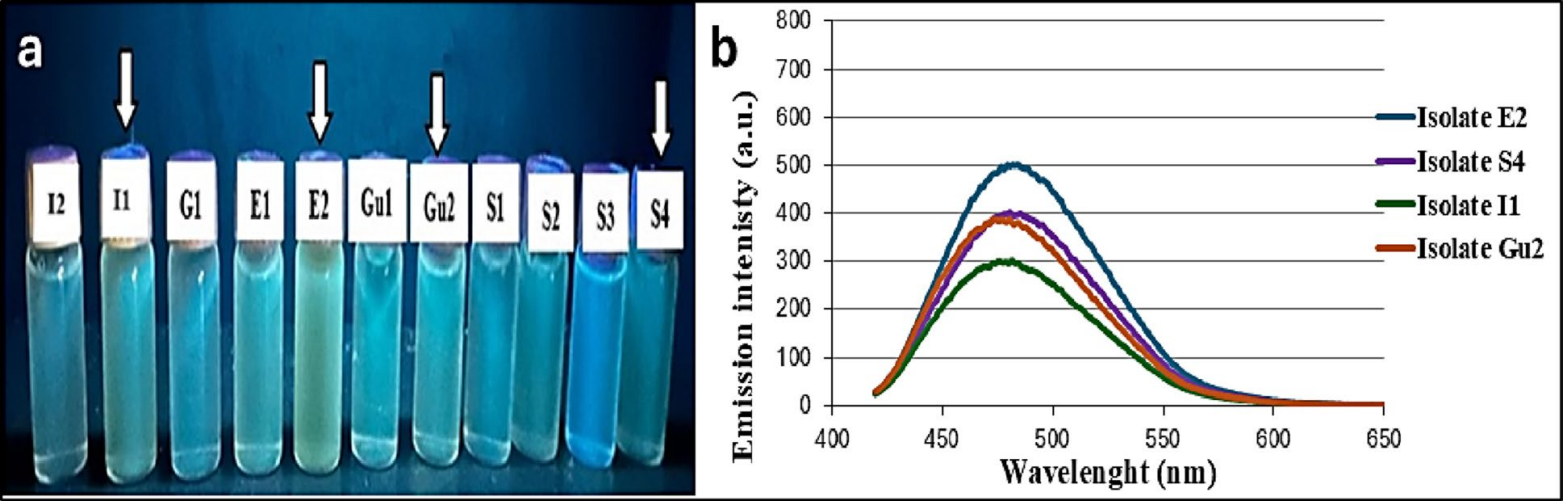
Fluorescence production by different bacterial isolates. (**a**) Under UV lamp at 390 nm. (**b**) Emission spectra using Luminescence Spectrometer

Primary screening of fluorescence by visual observation under UV illumination at 390 nm revealed that I1, E2, Gu2 and S4 isolates exhibted markedley higher fluorescence compared with other isolates (Fig. 3a). Subsequent quantitative secondary screening was based on fluorescence emission intensity which is directly proportional to the actual amount of GFP. The data presented in Fig. 3b confirmed that E2 isolate recovered from the squid’s eye exhibited a distinctive fluorescence intensity. The detection GFP production typically relies on measuring cellular fluorescence intensity, which correlates directly with the intracellular level of synthesized GFP, as reported by Korneli et al. [44].

### Selection of the most potent GFP-producing bacterial isolate

Although fluorescence intensity is a convenient indicator of GFP production, it doesn’t provide information about GFP purity as it is affected by background signals.it is not sufficient for selecting the most efficient GFP-producing isolate [45]. Therefore, protein-based analyses were employed to select the most efficient isolate for pure GFP production.

#### I. Total protein and GFP quantification

Total soluble protein and GFP concentrations were determined for the four selected isolates (E2, S4, I1, and Gu2) recovered from secondary screening. Quantitative analysis revealed that isolate E2 produced the highest GFP concentration (595.633 µg/ml) with high purity of 44.28% (Table 2), justifying its selection for subsequent optimization and purification steps.

#### II. Protein profiling using SDS-PAGE

**Fig. 4:**
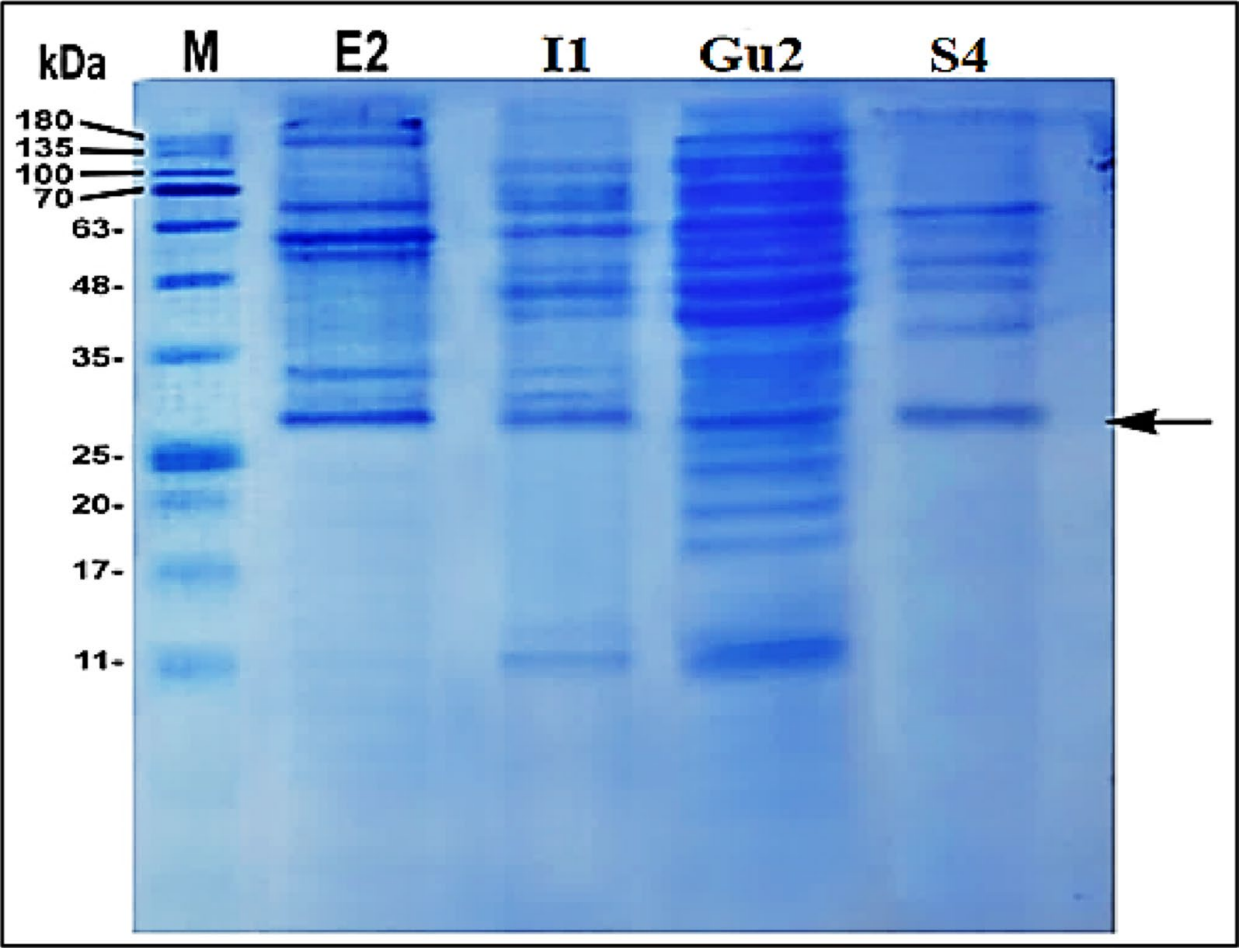
Electrophoretic pattern of total proteins from the selected bacterial isolates using SDS–PAGE. Lane M represents the molecular weight standards (kDa), E2,I1,Gu2, and S4 are the bacterial isolates. The band corresponding to GFP (~ 27 kDa) was indicated by an arrow

SDS-PAGE analysis revealed a distinct common protein band around 27 kDa in all extracted proteins from the four selected isolates (E2, I1, Gu2 and S4) (Fig. 4). E2 isolate exhibited a markedly more intense band, indicating a higher level of GFP content relative to the other. The original, high resolution gel images was provided as supplementary Fig. S1. Based on combined quantitative and qualitative protein analyses, isolate E2 was selected as the most potent GFP producer. Similar application of SDS–PAGE for comparative analysis of protein profiles to assess expression efficiency and solubility in bacterial systems has been reported [46].

**Table 2:** Total protein and green fluorescent protein concentrations for the selected fluorescent bacterial isolates

Bacteria	Total soluble Protein concentration (µg/ml)	GFP concentration (µg/ml)	GFP purity (%)
E2	1345.144	595.633	44.28
S4	951.821	280.352	29.4
I1	967.304	306.265	31.6
Gu2	1639.815	463.783	28.28

### Identification of the most potent GFP-producing bacterial isolate

#### Morphological characteristic

**Fig. 5:**
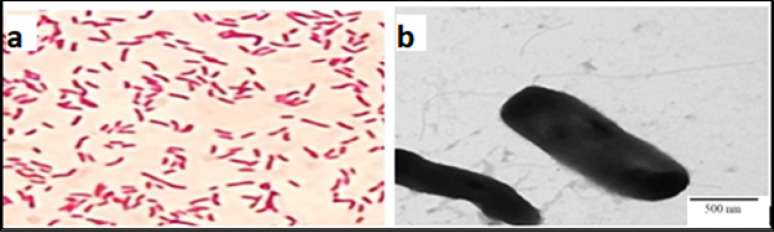
Microscopic characterization of E2 isolate. (**a**) Gram staining under light microscope at 1000X magnification. (**b**) Transmission electron micrograph at 12000X (scale bar = 500 nm)

Morphological examination revealed that the colony of the fluorescent bacterial isolate E2 is circular, medium- sized with entire margin, smooth texture, convex elevation and opaque appearance. Microscopic examination of E2 isolate (Fig. 5) revealed that it is gram negative, motile rod, and bipolarly flagellated bacterium.

### Biochemical profiling

The biochemical profile of the fluorescent bacterial isolate E2 summarized in Table 3, revealed distinctive metabolic and enzymatic characteristics that aided in the taxonomic identification of the isolate. E2 isolate exhibited a positive reaction for several key biochemical tests, including L-Pyrrolidonyl Arylamidase, D-glucose utilization, D-mannose assimilation, L-proline arylamidase, tyrosine arylamidase, urease, and coumarate assimilation. These positive results indicate the organism’s ability to metabolize specific amino acids, simple carbohydrates, and aromatic compounds, reflecting a versatile metabolic profile consistent with members of the Pseudomonas group. In contrast, negative results were observed for a wide range of tests, including β-N-Acetylglucosaminidase, lipase, H₂S production, citrate utilization, malonate assimilation, and glucose fermentation, suggesting that isolate E2 predominantly relies on oxidative rather than fermentative pathways for energy generation. Based on the comprehensive biochemical fingerprint obtained by the VITEK^®^ 2 system, E2 isolate was preliminary identified as *Pseudomonas fluorescens* with a high confidence level of 95% probability. This result is consistent with the phenotypic and molecular characteristics typically reported for this species, supporting its classification as a fluorescent Pseudomonas strain (Table 3).

**Table 3:** Biochemical profiling of E2 isolate using VITEK^®^ 2 automated system

Well	Test	Amount/Well (mg)	Result
2	Ala-Phe-Pro-Arylamidase	0.0384	−
3	Adonitol	0.1875	−
4	L-Pyrrolidonyl Arylamidase	0.018	+
5	L-Arabitol	0.3	−
7	D-Cellobiose	0.3	−
9	Beta-Galactosidase	0.036	−
10	H_2_S production	0.0024	-
11	β-N-Acetylglucosaminidase	0.0408	−
12	Glutamyl Arylamidase pNA	0.0324	−
13	D-Glucose	0.3	+
14	γ-Glutamyl Transferase	0.0228	−
15	Glucose fermentation	0.45	−
17	Beta-Glucosidase	0.036	−
18	D-Maltose	0.3	−
19	D-Mannitol	0.1875	−
20	D-Mannose	0.3	+
21	Beta-Xylosidase	0.0324	−
22	Beta-Alaninearylamidase pNA	0.0174	−
23	L-Proline Arylamidase	0.0234	+
26	Lipase	0.0192	−
27	Palatinose	0.3	−
29	Tyrosine Arylamidase	0.0276	+
31	Urease	0.15	+
32	D-Sorbitol	0.1875	−
33	Sucrose	0.3	−
34	D-Tagatose	0.3	−
35	D-Trehalose	0.3	−
36	Citrate (Sodium)	0.054	−
37	Malonate	0.15	−
39	5-Keto-D-Gluconate	0.3	−
40	L-Lactate alkalinization	0.15	−
41	α-Galactosidase	0.036	−
42	Succinate alkalinization	0.15	−
43	β-N-Acetylgalactosaminidase	0.0306	−
44	Alpha-Galactosidase	0.036	−
45	Phosphatase	0.0504	−
46	Glycine Arylamidase	0.012	−
47	Ornithine Decarboxylase	0.3	−
48	Lysine Decarboxylase	0.15	−
52	Decarboxylase Base	N/A	−
53	L-Histidine assimilation	0.087	−
56	Coumarate	0.126	+
57	β-Glucuronidase	0.0378	−
58	O/129 Resistance (comp. *Vibrio*)	0.0105	−
59	Glu-Gly-Arg-Arylamidase	0.0576	−
61	L-Malate assimilation	0.042	−
62	Ellman Test (Sulfhydryl Compounds)	0.03	−
64	L-Lactate assimilation	0.186	−

### Molecular identification of the most potent fluorescent bacteria

The most potent fluorescent isolate, E2, was identified by analyzing its 16 S rRNA gene sequence. The obtained sequence was subjected to comparison with sequences in the GenBank repository using BLASTN to identify its closest phylogenetic relatives. BLAST analysis revealed that isolate E2 shared a 99.34% sequence similarity with *Pseudomonas gessardii* over an aligned region of 604 base pairs, indicating a strong genetic affiliation with this species. Strain E2’s 16 S rRNA gene sequence has been submitted to the GenBank database and is available under the accession number OM979087.

**Fig. 6:**
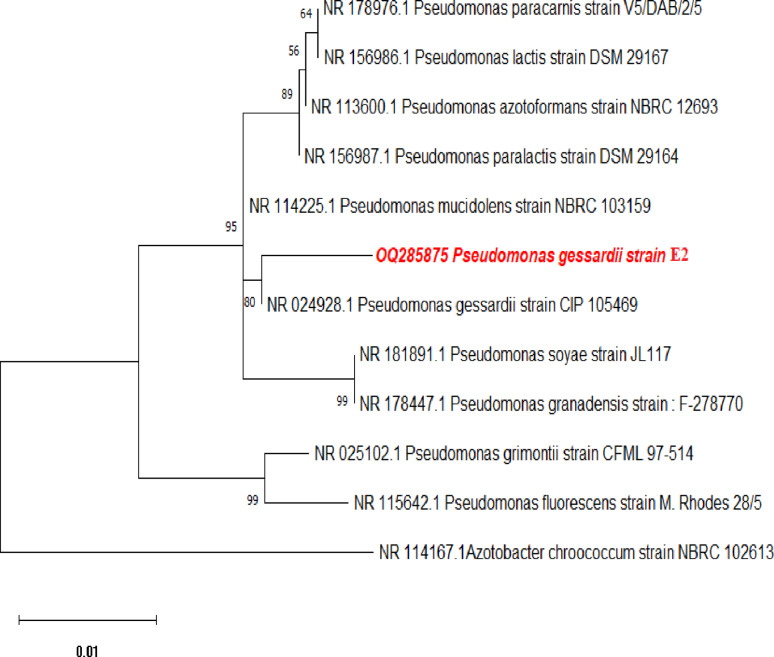
Neighbor-Joining tree constructed from nearly complete 16 S rRNA sequences showing the clustering of strain E2 with type strains of closely related Pseudomonas species. Bootstrap support values in % derived from 1000 replicates are indicated at nodes. The scale bar corresponds to 0.01 substitutions per nucleotide position, with only values exceeding 50% presented

To further confirm the taxonomic position of the isolate, a phylogenetic tree was generated using E2 strain’s 16 S rRNA gene sequences along with those of its closest Pseudomonas species. The phylogenetic analysis demonstrated that strain E2 clustered within the *Pseudomonas gessardii* clade, forming a distinct phyletic lineage that strongly supported by a high bootstrap score (1000 replicates), thereby confirming its close evolutionary relationship with *P. gessardii* (Fig. 6).

### Optimization of physical factors affecting GFP production by *Pseudomonas gessardii* using OFAT strategy

**Fig. 7:**
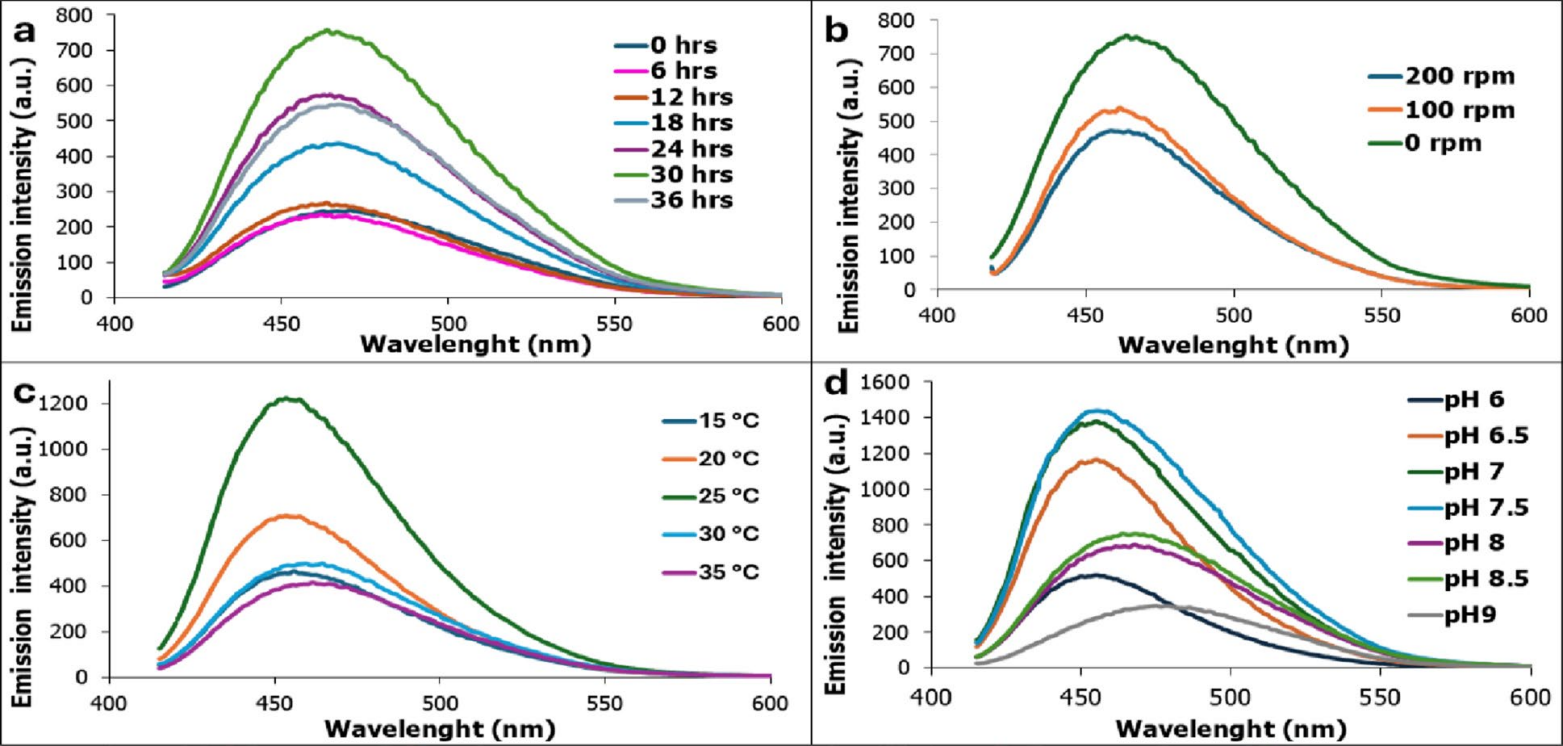
Effect of the physical conditions on the fluorescence spectrum of green fluorescent protein produced by *Pseudomonas gessardii*. (**a**) Incubation period (h). (**b**) Incubation state( rpm). (**c**) Incubation temperature (ºC). (**d**) pH

**Fig. 8:**
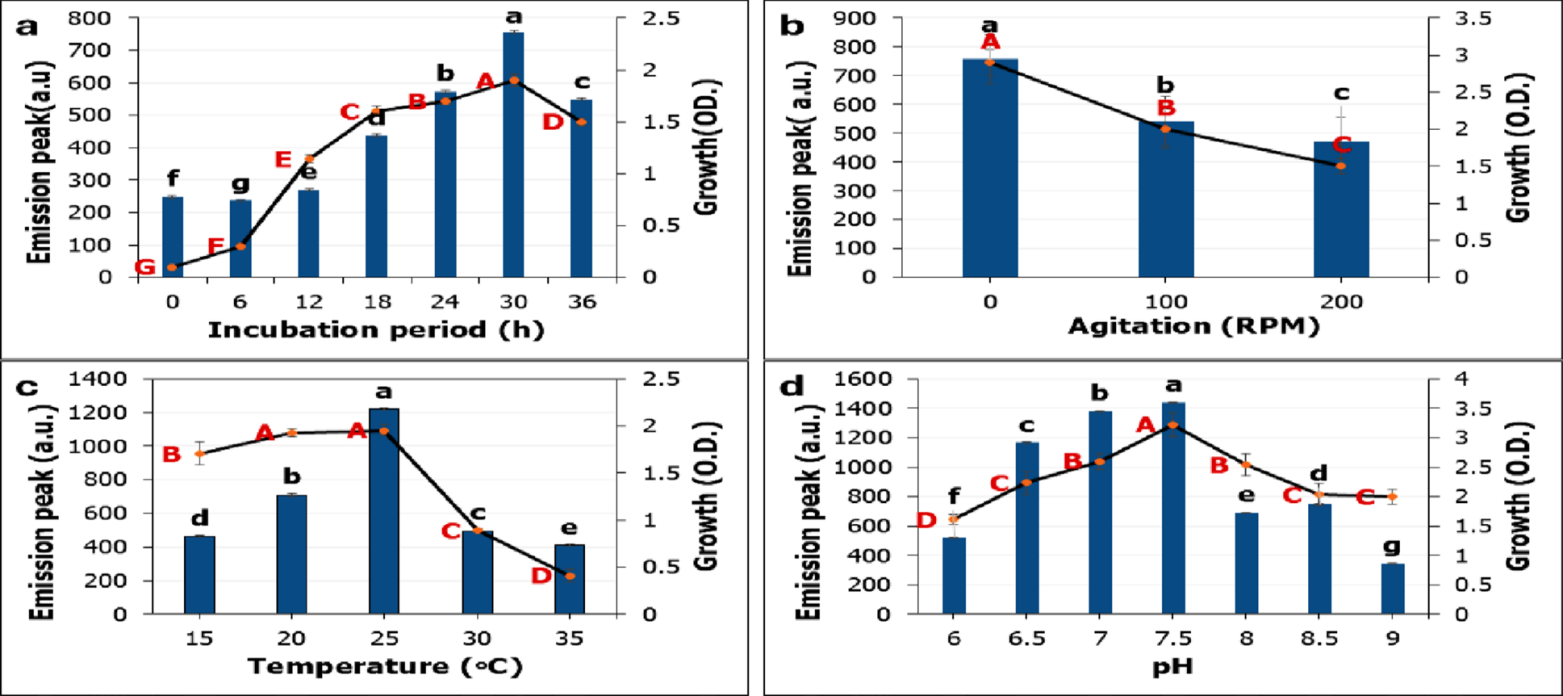
Emission peaks (a.u) of green fluorescence protein and growth of *Pseudomonas gessardii*. at different physical factors. (**a**) Incubation period. (**b**) incubation state. (**c**) incubation temperature. (**d**) pH. Error bars represent standard deviations. Columns and curve points labeled with distinct letters represent statistically significant differences

The fluorescence spectrum profiles presented in Fig. 7 demonstrate the physical factors like incubation period, agitation, incubation temperature, and pH affect directly on the production of GFP by *Pseudomonas gessardii.* Across all tested factors, the emission spectra of GFP retain a characteristic peak at 453–470 nm range indicating the changes in fluorescence intensity may be due to differential chromophore or protein expression rather than alterations in protein structure. GFP emission peak and *P. gessardii* growth increased progressively from 0 to 30 h incubation time, with the highest emission recorded at 30 h. This trend suggests that GFP production is tightly coupled with active cellular metabolism (Fig. 8a). Fluorescence phenomenon is generally acknowledged as being dependent on cell density, wherein fluorescent organisms exhibit maximum glow until reaching a threshold population density, after which further increases in cell density result in a decline in fluorescence [52].

Oxygen limitation enhance growth and GFP production by *P. gessardii*. Static condition at 0 rpm recorded the highest emission peak (Fig. 8b). This may reflect a suppression of GFP biosynthesis and bacterial growth under high oxygen transfer rates. Additionally, static conditions may favor the microaerophilic or low-oxygen environment required for bacterial growth and GFP production which encourage the application of *P. gessardii* GFP for mapping tumor microenvironment. Similar report was by Nealson and Hastings [47] who recorded the maximum luminescence production was at low oxygen level. However, Abdel-Hamid and Blaghen [48] reported that the emission of luminescence by *Vibrio fischeri* requires proper aeration and agitation conditions.

Temperature and pH are critical factors and need to be carefully regulated, particularly for biosynthetic processes that involve enzymes that are sensitive to pH or temperature. Cultivation of *P. gessardii* at 25 °C supported the high yield of GFP and growth (Fig. 8c) further elevation in temperature beyond 30 °C resulted in decline. It was established that the production of GFP was enhanced at lower temperature. Perez-Arellano, & Perez-Martinez [49] reported 30 °C as the optimum for GFP by *Lactobacillus casei.* Higher incubation temperature at 37 °C resulted in unfunctional non fluorescent protein which may be due to improper folding [50]. Production of GFP is very sensitive to pH of culture media. pH of 7.5 recorded the highest significant results for growth and GFP (Fig. 8d) This indicates that *P. gessardii* exhibits a preference for a neutral to slightly alkaline pH environment. Moreover, the emission spectra of GFP are distinguishable at different pHs which may be due to chromophore protonation ( Fig. 7d). Yeung et al. [51] recorded that GFP can only fluoresces at pH ranged from 6.5–10.At pH 4,fluorescence is completely absent due to the protonation of chromophore amino acids. In addition, Smith et al. [52] reported the highest emission intensity of GFP expressed in *Bacillus subtilis* at pH 7 and decreased at both acidic and alkaline conditions.

**Fig. 9:**
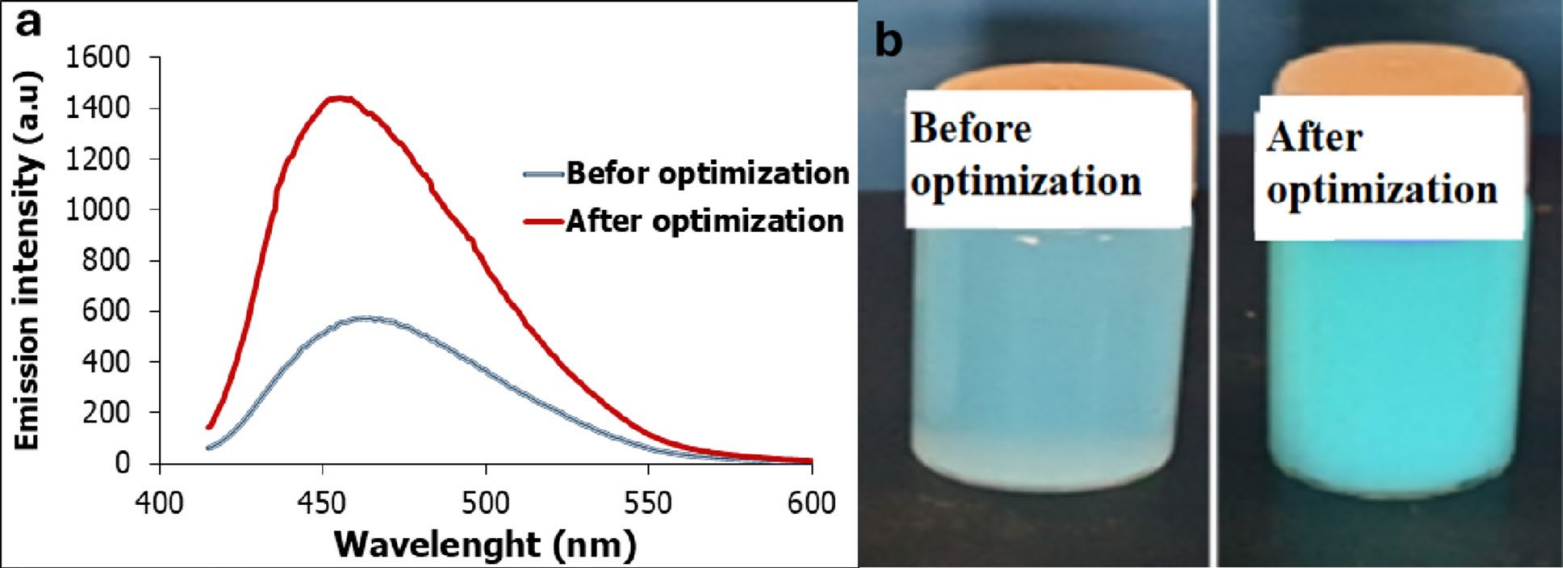
Effect of physical factors optimization on green fluorescence protein produced by *Pseudomonas gessardii*. (**a**) Emission intensity (a.u) of GFP. (**b**) Fluorescence emission under UV illumination at 365 nm

Collectively, optimization of environmental parameters resulted in a remarkable improvement in GFP fluorescence intensity by 1.51 fold compared to unoptimized conditions, demonstrating the effectiveness of the applied strategy (Fig. 9). Similarly, Chew et al. [50] demonstrated that optimized growth conditions were found to boost functional GFP production nearly nine times higher than that achieved under unoptimized conditions.

### Purification and validation of GFP

GFP was purified using a sequential strategy combining ATPS with HIC. ATPS is a well-established liquid–liquid extraction technique that is efficient, scalable, environmentally sustainable, and biocompatible, commonly applied in protein purification processes including GFP [34]. GFP in the cell free supernatant of optimized culture of *P.gessardii* (Fig S2a) was purified according to ATPS procedures which involves two steps. Firstly, GFP was extracted in ethanol layer in which the fluorescence was detected under UV illumination (Fig S2b).Secondly, butanol was added as less polar and more hydrophobic solvent, enabling GFP to partition into the bottom aqueous phase (Fig S2c). This process provided further purification and increased GFP concentration [41]. Dialysis step was also performed to concentrate the partially purified GFP and get rid of any organic solvents and residual salts that may denature GFP (Fig S2d).

**Fig. 10:**
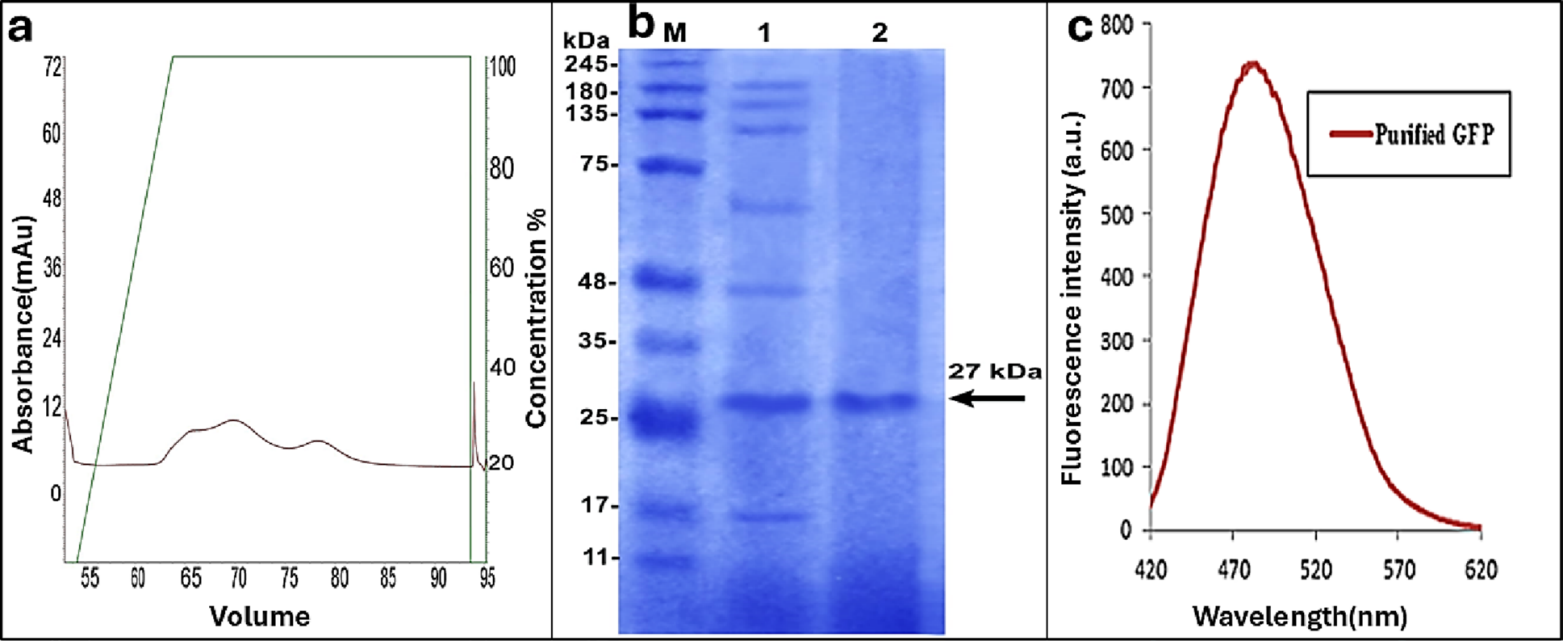
Purification and characterization of *P.gessardii*. GFP using Fast Protein Liquid Chromatography. (**a**) The elution curve of GFP using hydrophobic interaction chromatography (HIC) at 365 nm. (**b**) GFP characteristic band on SDS-PAGE. Lane M: protein ladder in kDa; Lane 1: cell free filtrate of *P.gessardii* culture; Lane 2: GFP band after purification. (**c**) Fluorescence emission spectrum of purified GFP

Further purification using HIC revealed a single, dominant, well defined, and sharp peak eluted at approximately 93 ml (Fig. 10a). SDS-PAGE analysis confirmed the presence of single protein band at approximately 27 kDa corresponding to GFP (Fig. 10b), indicating high purity. The original raw SDS-PAGE gel image was provided as supplementary Fig. S3. The purified GFP showed a characteristic emission peak at 485 nm after excitation at 395 nm (Fig. 10c). Overall, the applied purification workflow resulted in high quality GFP achieving 96.3% purity and recovery yield of 53.03% (Table 4). These results are considered satisfactory when compared to the 34.1% recovery of recombinant GFP reported by Dong et al. [34].

**Table 4:** Purification of GFP produced by *P.gessardii*

Purification stage	Total soluble protein(µg/ml)	GFP (µg/ml)	Purity %	GFP yield %
Crude supernatant	1115.16	595.72	53.61	100
HIC column	328.04	315.92	96.3	53.03

## Conclusion

Since the initial recovery of GFP from jellyfish *Aequorea Victoria*, no confirmed reports have described the isolation of native GFP directly from bacterial sources. Most results relies exclusively on heterologous expression in prokaryotic systems. In the current study, *Pseudomonas gessardii* E2 strain represented as a novel and efficient source of high quality GFP. Optimization of key environmental parameters significantly improved the fluorescence emission and production of GFP. Moreover, the applied purification strategy successfully yielded high purified GFP confirming the effectiveness and suitability of the downstream process for recovery and purification of marine-derived GFP. In conclusion, these results introduce a new insights of marine bacteria as alternative, novel, and renewable source of GFP for bioimaging applications.

Despite these promising findings, the study is subjected to certain limitations. The identification of GFP was primarily based on molecular weight, fluorescence characteristics, and chromatographic behavior, while advanced proteomic confirmation techniques such as MALDI-TOF mass spectrometry were not employed. Therefore, further structural, proteomic, and genomic investigations are warranted to fully elucidate the molecular basis of GFP biosynthesis in *P. gessardii*. Future studies should also explore genetic characterization and scalability to assess the feasibility of large-scale production and broader biotechnological and environmental applications.

## Supplementary Information

Below is the link to the electronic supplementary material.

Supplementary Material 1

## Data Availability

No datasets were generated or analysed during the current study.

## Abbreviations

GFP: Green fluorescent protein
SWC: Sea Water Complete agar
kDa: kilodaltons
LM: Luminescent medium
SDS–PAGE: Sodium dodecyl sulfate polyacrylamide gel electrophoresis
BLASTN: Basic Local Alignment Search Tool
OFAT: One-factor-at-a-time strategy
ATPS: Aqueous two-phase system
FPLC: Fast Protein Liquid Chromatography

## References

[1] Whitcher C, Ron SR, Ayala-Varela F, Crawford AJ, Herrera-Alva V, Castillo-Urbina EF, et al. Evidence for ecological tuning of Anuran biofluorescent signals. Nat Commun. 2024;15(1):1–17. 39406728 10.1038/s41467-024-53111-w PMC11480117

[2] Kohler AM, Olson ER, Martin JG, Anich PS. Ultraviolet fluorescence discovered in new world flying squirrels (Glaucomys). J Mammal. 2019;100:21–30.

[3] Prasher DC, Eckenrode VK, Ward WW, Prendergast FG, Cormier MJ. Primary structure of the aequorea Victoria green-fluorescent protein. Gene. 1992;111(2):229–33. 1347277 10.1016/0378-1119(92)90691-h

[4] Anter HA, Husseiny SM, Mohamed FA. Green fluorescent protein: discover, structure, purification, applications, and future prospects. J Sci Res Sci. 2023;40:53–71.

[5] Kumar A, Pal D. Green fluorescent protein and their applications in advance research. J Res Eng Appl Sci. 2016;01(01):42–6.

[6] Li JJ, Wang AQ, Janson JC, Ballagi A, Chen J, Liu YD, et al. Immobilized triton X-100-assisted refolding of green fluorescent Protein-Tobacco etch virus protease fusion protein using β-cyclodextrin as the eluent. Process Biochem. 2009;44(3):277–82.

[7] Wouters FS, Verveer PJ, Bastiaens PIH. Imaging biochemistry inside cells. Trends Cell Biol. 2001;11(5):203–11. 11316609 10.1016/s0962-8924(01)01982-1

[8] Hoffman RM. Application of GFP imaging in cancer. Lab Investig. 2015;95(4):432–52. 25686095 10.1038/labinvest.2014.154 PMC4383682

[9] Hirano M, Ando R, Shimozono S, Sugiyama M, Takeda N, Kurokawa H, et al. A highly photostable and bright green fluorescent protein. Nat Biotechnol. 2022;40(7):1132–42. 35468954 10.1038/s41587-022-01278-2 PMC9287174

[10] Cubitt A, Heim R, Adams SR, Gross EA, Tsien La. Understanding, improving and using green fluorescent proteins. Trends Biochem Sci. 1995;20(11):448–55. 8578587 10.1016/s0968-0004(00)89099-4

[11] Heim R, Prasher DC, Tsien RY. Wavelength mutations and posttranslational autoxidation of green fluorescent protein. Proc Natl Acad Sci U S A. 1994;91(26):12501–4. 7809066 10.1073/pnas.91.26.12501 PMC45466

[12] Kannahi M, Sivasankari S. Isolation and identification of bioluminescent bacteria from marine water at Nagapattinam sea shore area. Int J Pharm Sci Rev Res. 2014;26(2):346–51.

[13] Moussa H, Ibrahem AB, El-Sayed A, Mohammed F. In vitro evaluation of anti-microbial activities of marine streptomyces against viral models, bacterial and fungal strains. Int J Virol. 2015;11(1):20–31.

[14] Bayyana S, Yalla SK, Cherian T, Mohanraju R. Isolation and characterization of bioluminescent bacteria associated with uroteuthis Duvaucelli, an Indian squid from Andaman waters. Glob J Biosci Biotechnol. 2018;7:353–8.

[15] Harvey EN. A history of luminescence from the earliest times until 1900. Vol. 44, physics today. Philadelphia: American Philosophical Society; 1957. p. 692.

[16] Salvà-Serra F, Nimje P, Piñeiro-Iglesias B, Alarcón LA, Cardew S, Inganäs E, et al. Description of Pseudomonas Imrae sp. nov., carrying a novel class C β-lactamase gene variant, isolated from gut samples of Atlantic mackerel (Scomber scombrus). Front Microbiol. 2025;16:1–13. 10.3389/fmicb.2025.1530878 PMC12057487 40336828

[17] Verhille S, Batda N, Dabboussi F, Hamze M, Izard D, Leclerc H et al. Pseudomonas gessardii sp. nov. and Pseudornonas migulae sp. nov., two new species isolated from natural mineral waters. Int J Syst Bacteriol. 1999;49:1559–72. 10.1099/00207713-49-4-1559 10.1099/00207713-49-4-1559 10555337

[18] Ebraheem MA, El-Fakharany EM, Husseiny SM, Mohammed FA. Purification and characterization of the produced hyaluronidase by Brucella intermedia MEFS for antioxidant and anticancer applications. Microb Cell Fact. 2024;23(1):1–15. 39026213 10.1186/s12934-024-02469-z PMC11256544

[19] Mohamed Korayem AS, Mohammed FA, Abu-Hussien SH, Abosamra FM, El-Dein SN, Rahmy HAF. Statistical optimization of Solid-State fermentation by Aspergillus oryzae for valorization of Olive cake and its application as a poultry feed. Scientifica (Cairo). 2025;2025(1):1–13.

[20] Liu JL, Lin SP, Nguyen TDH, Panjapornpon C, Prapainainar P, Jitapunkul K, et al. Integrated strategy for effecient production and direct purification of EGFP from unclarified feedstocks. Biochem Eng J. 2025;222:109822.

[21] Niswender KD, Blackman SM, Rohde L, Magnuson AM, Piston DW. Quantitative imaging of green fluorescent protein in cultured cells: comparison of microscopic techniques, use in fusion proteins and detection limits. J Microsc. 1995;180:109–25. 8537958 10.1111/j.1365-2818.1995.tb03665.x

[22] Bolelli L, Ferri EN, Girotti S. The management and exploitation of naturally light-emitting bacteria as a flexible analytical tool: A tutorial. Anal Chim Acta. 2016;934:22–35. 27506340 10.1016/j.aca.2016.05.038

[23] Atlas RM. Handbook of microbiological media. Fourth Edi. Atlas RM, editor. CRC Press; 2010. 2036 p.

[24] Reichelt JL, Baumann P. Taxonomy of the marine, luminous bacteria. Arch Mikrobiol. 1973;94(4):283–330.

[25] Badar U, Shoeb E, Daredia K, Shawar De, Akhtar J, Ansari MA. Screening and characterization of luminescent bacterial strain. J Basic Appl Sci. 2012;8(2):602–6.

[26] Naguit MAA, Plata KC, Abisado RG, Calugay RJ. Evidence of bacterial bioluminescence in a Philippine squid and Octopus hosts. AACL Bioflux. 2014;7(6):497–507.

[27] Yaser NA, Faiz M, Abdullah F, Aris AM, Zainudin II. Isolation and identification of bioluminescent bacteria in squid and water of Malaysia. Int J Adv Agric Environ Eng. 2015;1(2). 225-8. 10.15242/IJAAEE.C0215136

[28] Alias R, Mohtar SH, Yunus YM, Saadun R. Isolation and identification of luminescent glowing bacteria in. J Built Environ Technol Eng. 2017;3:58–65.

[29] Balan SS, Raffi SM, Jayalakshmi S. Probing of potential luminous bacteria in Bay of Bengal and its enzyme characterization. J Microbiol Biotechnol. 2013;23(6):811–7. 23676923 10.4014/jmb.1206.06020

[30] Jain S, Teotia S, Gupta MN. Purification of green fluorescent protein overexpressed by a mutant Recombinant Escherichia coli. Protein Expr Purif. 2004;36(1):76–81. 15177287 10.1016/j.pep.2004.04.008

[31] Haberl Meglič S, Janež N, Peterka M, Flisar K, Kotnik T, Miklavčič D. Evaluation and optimization of protein extraction from E. coli by electroporation. Front Bioeng Biotechnol. 2020;8:543187. 33015013 10.3389/fbioe.2020.543187 PMC7506034

[32] Bradford MM. A rapid and sensitive method for the quantitation of microgram quantities of protein utilizing the principle of protein-dye binding. Anal Biochem. 1976;72(1–2):248–54. 942051 10.1016/0003-2697(76)90527-3

[33] Cohen SL, Chait BT. Mass spectrometry of whole proteins eluted from SDS-PAGE gels. Anal Biochem. 1997;247:257–67. 9177686 10.1006/abio.1997.2072

[34] Dong J, Ding X, Wang S. Purification of the Recombinant green fluorescent protein from tobacco plants using alcohol/salt aqueous two-phase system and hydrophobic interaction chromatography. BMC Biotechnol. 2019;19(1):1–8. 31818280 10.1186/s12896-019-0590-y PMC6902424

[35] Aneja KR. Experiments in microbiology plant pathology and biotechnology. 4th edn. New Age International; 2003. 632 p.

[36] Aygan A, Arikan B. Mini review an overview on bacterial motility detection. Int J Agri̇culture &Bi̇ology. 2007;9(1):193–6.

[37] Hoeniger JFM. Development of flagella by proteus rnirabilis. J Gen Microbiol. 1964;40:29–42.

[38] Nayyar C, Thakur P, Tak V, Saigal K. Stenotrophomonas maltophilia: an emerging pathogen in paediatric population. J Clin Diagn Res. 2017;11(1):8–11. 10.7860/JCDR/2017/24304.9318 PMC5324411 28273966

[39] Altschul SF, Madden TL, Schäffer AA, Zhang J, Zheng Z, Miller W, et al. Gapped BLAST and PSI-BLAST: a new generation of protein database search programs. Nucleic Acids Res. 1997;25:3389–402. 9254694 10.1093/nar/25.17.3389 PMC146917

[40] Felsenstein J. Confidence limits on phylogenies: an approach using the Bootstrap. Evolution (N Y). 1985;39(4):783–91. 10.1111/j.1558-5646.1985.tb00420.x 28561359

[41] Samarkina ON, Popova AG, Gvozdik EY, Chkalina AV, Zvyagin IV, Rylova YV, et al. Universal and rapid method for purification of GFP-like proteins by the ethanol extraction. Protein Expr Purif. 2009;65(1):108–13. 19084068 10.1016/j.pep.2008.11.008

[42] Parmar P, Shukla A, Saraf M, Patel B. Isolation of bioluminescent bacteria from marine organisms. Indian J Geo-Mar Sci. 2020;49(3):471–6.

[43] Dubey PA, Sharon M. Culture parameters affect the light emitting property of organisms isolated from two marine fishes. Acta Sci Microbiol. 2020;3(5):55–61.

[44] Korneli C, David F, Biedendieck R, Jahn D, Wittmann C. Getting the big Beast to work—Systems biotechnology of Bacillus megaterium for novel high-value proteins. J Biotechnol. 2013;163:87–96. 22750448 10.1016/j.jbiotec.2012.06.018

[45] Hunga CW, Holomana TRP, Kofinas P, Bentley WE. Towards oriented assembly of proteins onto magnetic nanoparticles. Biochem Eng J. 2008;38:164–70. 10.1016/j.bej.2007.06.017

[46] Crowe J, Bradshaw T, Monk P. Chemistry for the Biosciences. The essential concepts. Oxford; New York: Oxford University Press; 2006. p. 571.

[47] Nealson KH, Platt T, Hastings JW. Cellular control of the synthesis and activity of the bacterial luminescent system. J Bacteriol. 1970;104(1):313–22. 5473898 10.1128/jb.104.1.313-322.1970 PMC248216

[48] Abdel-Hamid AA, Blaghen M. Optimization of bioluminescence of vibrio fischeri and assessment of Hg++, Cd++, As++, Zn++, Ag+, Cu + + and Ni + + Ions. Asian J Biotechnol Bioresour Technol. 2018;4(2):1–9.

[49] Pérez-Arellano I, Pérez-Martínez G. Optimization of the green fluorescent protein (GFP) expression from a lactose-inducible promoter in Lactobacillus casei. FEMS Microbiol Lett. 2003;222(1):123–7. 12757955 10.1016/S0378-1097(03)00244-1

[50] Chew FN, Tan WS, Boo HC, Tey BT. Statistical optimization of green fluorescent protein production from Escherichia coli BL21(DE3). Prep Biochem Biotechnol. 2012;42(6):535–50. 23030465 10.1080/10826068.2012.660903

[51] Yeung T, Touret N, Grinstein S. Quantitative fluorescence microscopy to probe intracellular microenvironments. Curr Opin Microbiol. 2005;8(3):350–8. 15939361 10.1016/j.mib.2005.04.004

[52] Smith CB, Anderson JE, Fischer RL, Webb SR. Stability of green fluorescent protein using luminescence spectroscopy: is GFP applicable to field analysis of contaminants? Environ Pollut. 2002;120(3):517–20. 12442776 10.1016/s0269-7491(02)00227-0

