# Supplementary material for: Marine-derived Pseudomonas gessardii E2 strain: a novel source of green fluorescent protein with insights into its optimization and purification
Source: Microb Cell Fact. 2026 Mar 11;25:89. doi: 10.1186/s12934-026-02960-9 (PMC13064211; doi:10.1186/s12934-026-02960-9)
Supplement: Supplementary file 1 — Supplementary Material 1 [file 12934_2026_2960_MOESM1_ESM.pdf]

Marine-derived *Pseudomonas gessardii* E2 strain: A novel source of green fluorescent protein with insights into its optimization and purification.

Fafy A. Mohammed[1], Hend A. Anter [1], Sherif Moussa Husseiny[1]

[1] Botany department, Faculty of Women For Arts, Science and Education, Ain Shams University, Egypt; Fafy.AbdEl-Rahman@women.asu.edu.eg.,hendantar@women.asu.edu.eg, husseinymoussa@women.asu.edu.eg,

*Correspondence: Fafy.AbdEl-Rahman@women.asu.edu.eg. , Tel.: +201064880850

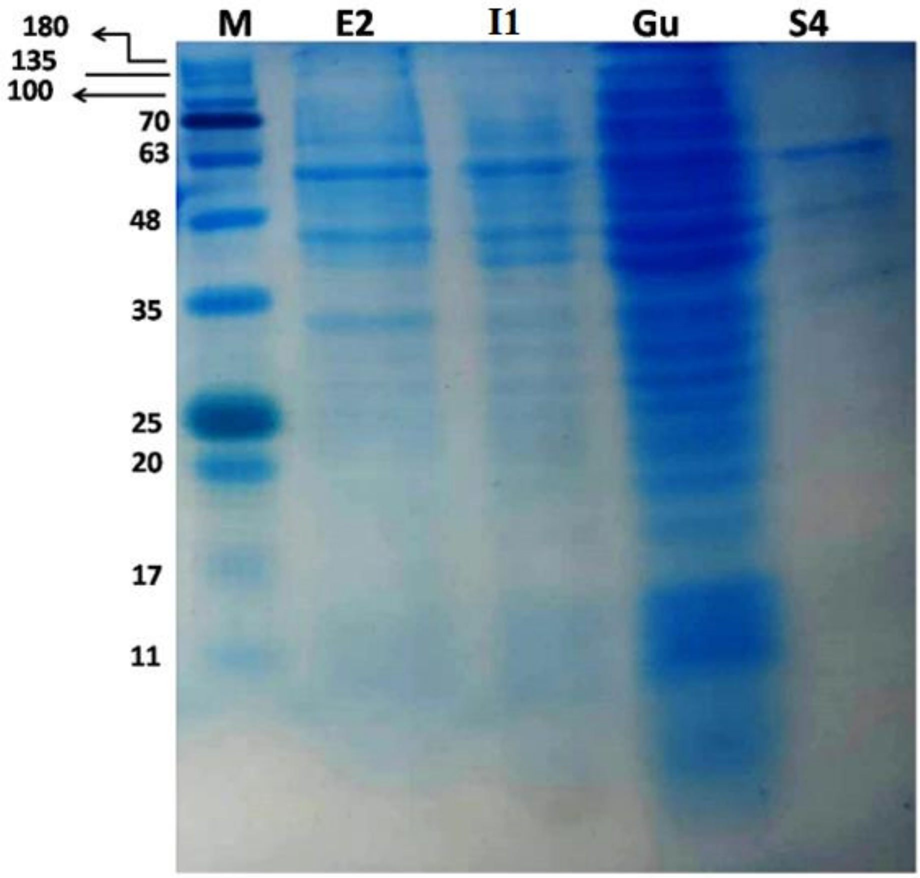

Fig S1 Original uncropped SDS–PAGE gel image showing the total protein profiles of selected fluorescent bacterial isolates (E2, I1, Gu2, and S4). The gel reveals a distinct protein band at approximately 27 kDa, corresponding to the expected molecular weight of green fluorescent protein (GFP).

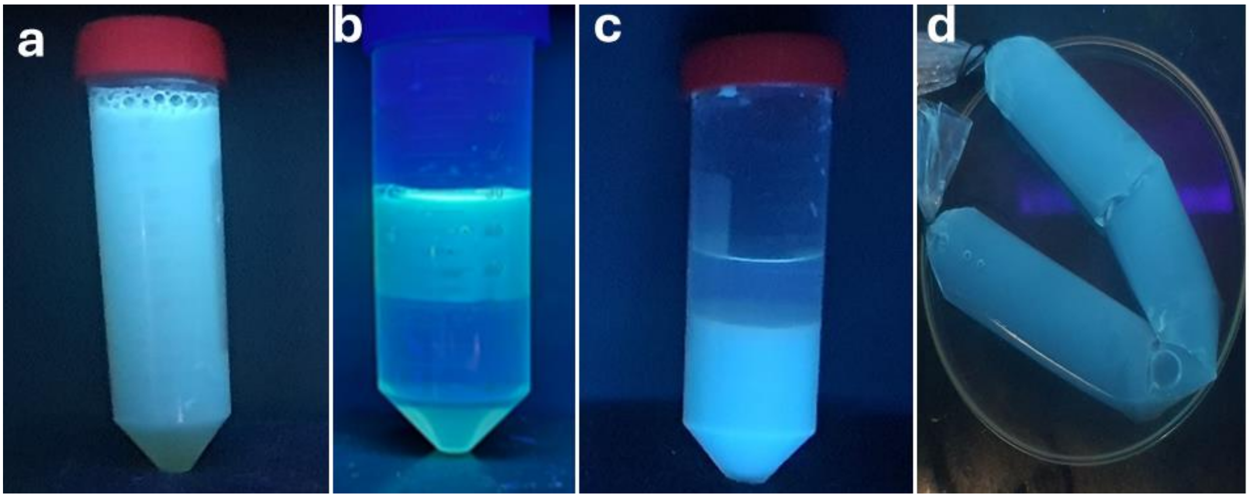

Fig. S2 Purification procedures of GFP using ATPS under UV illumination at 365nm.(a) The supernatant of *P.gessardii* culture after centrifugation at 6000rpm for 20min at 4 ºC.(b) The upper layer of GFP after extraction with ethanol.(c)The lower aqueous layer of GFP after *n*-butanol addition.(d) The dialysis bag enclosed GFP.

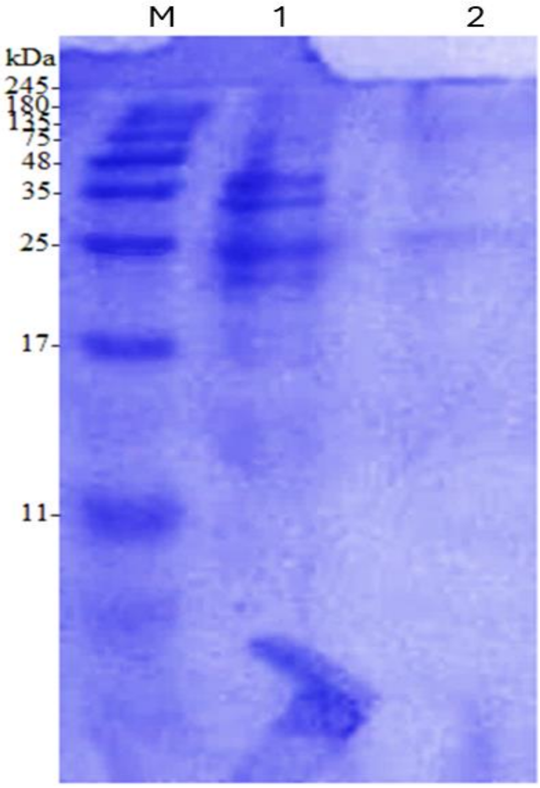

Fig S3 Original uncropped SDS–PAGE image showing GFP characteristic band; Lane M, protein ladder in kDa. Lane 1, cell free filtrate of *P.gessardii* culture. Lane 2, GFP band after purification.
